# *TGF-β/VEGF-A* Genetic Variants Interplay in Genetic Susceptibility to Non-Melanocytic Skin Cancer

**DOI:** 10.3390/genes13071235

**Published:** 2022-07-13

**Authors:** Letizia Scola, Maria Rita Bongiorno, Giusi Irma Forte, Anna Aiello, Giulia Accardi, Chiara Scrimali, Rossella Spina, Domenico Lio, Giuseppina Candore

**Affiliations:** 1 Clinical Pathology, Department of Bio-Medicine, Neuroscience, and Advanced Diagnostics, University of Palermo, 90135 Palermo, Italy; letizia.scola@unipa.it; 2 Section of Dermatology, Department of Health Promotion, Mother and Child Care, Internal Medicine and Medical Specialties, University of Palermo, 90127 Palermo, Italy; mariarita.bongiorno@unipa.it; 3 Institute of Bioimaging and Molecular Physiology, National Research Council (IBFM-CNR), 90015 Cefalù, Italy; giusi.forte@ibfm.cnr.it; 4 General Pathology, Department of Bio-Medicine, Neuroscience, and Advanced Diagnostics, University of Palermo, 90135 Palermo, Italy; anna.aiello@unipa.it (A.A.); giulia.accardi@unipa.it (G.A.); giuseppina.candore@unipa.it (G.C.); 5 Department of Health Promotion, Mother and Child Care, Internal Medicine and Medical Specialties, University of Palermo, 90127 Palermo, Italy; chiara.scrimali@unipa.it (C.S.); rosella.spina@unipa.it (R.S.); 6 Interdepartmental Research Center “Migrate”, University of Palermo, 90135 Palermo, Italy

**Keywords:** *TGF-β receptor genes*, *rs1800629*, *VEGF-A gene*, *rs3025039*, non melanocytic skin cancer, skin basal cell carcinoma, skin squamous cell carcinoma, genetic susceptibility

## Abstract

Differential genetically determined expression of transforming growth factor-β (TGF-β pathway and of vascular endothelial growth factor-A (VEGF-A) might modulate the molecular “milieu” involved in the etio-pathogenesis of non-melanoma skin cancer (NMSC). We have evaluated the frequency of some functionally relevant SNPs of *TGF-β* and *VEGF-A* genes in 70 NMSC patients and 161 healthy controls, typed for *TGF-β1 rs1800471*, *TGF-β2 rs900*, *TGF-βR1 rs334348* and *rs334349*, *TGF-βR2 rs4522809* and *VEGF-A rs3025039* SNPs. *TGF-βR2 rs1800629G* allele and related genotypes were found to be associated with a possible protective role against NMSC, whereas *VEGF-A rs3025039T* was associated with an increased risk. To evaluate the effect of genotype combinations on NMSC susceptibility, we determined the frequencies of 31 pseudo-haplotypes due to non-random linkage among alleles of loci not lying on the same chromosome. Two pseudo-haplotypes that imply a minor allele of *TGF-βR2* or minor allele of *VEGF-A* SNPs combined with major alleles of the other SNPs were, respectively, associated with a protective effect, and susceptibility to NMSC. In addition, a pseudo-haplotype involving minor alleles of *TGF-β2 rs900*, *TGF-βR1 rs334348* and *rs4522809* SNPs might be a susceptibility marker for NMSC. In conclusion, our data suggest that a complex interplay among the genetic polymorphisms of *TGF-β*, *TGF-β* receptors and *VEGF-A* genes might influence the net effect of genetic background of the patients on NMSC development. This might be relevant in the risk evaluation, diagnosis and treatment of NMSC.

## 1. Introduction

The incidence of the vast majority of cancers, including melanoma, increases with advancing age. However, the mechanism by which aging influences the risk of developing cancer is not fully understood. Whether this is attributable to declines in immunity or other changes, such as changes in the aging tissue microenvironment, remains unclear [1].

Non-melanoma skin cancers (NMSC) include basal cell carcinoma (BCC) and squamous cell carcinoma (SCC), which are responsible for approximately 80 and 20% of all NMSC cases, respectively [2,3]. In humans, skin cancer arises in the epidermis or hair bulbs where proliferating basaloid tumor cells are detected. The extension and severity of these phenomena, rate of skin aging and age of cancer appearance have a broad spectrum of inter-individual variability that might depend on different factors including the genetic background of each individual [4].

In particular, variants of the genes coding for molecules involved in the inflammatory response, mainly single nucleotide polymorphisms (SNP), can generate pro-inflammatory genotypes, which modulate and increase the chronic inflammation, and, in turn determining the formation of a molecular “milieu” that could accelerate the skin aging process and enhance the development and progression of cancer [5,6]. One fundamental signaling pathway with essential roles in tissue homeostasis is the transforming growth factor-β (TGF-β) pathway. TGF-β is a family of pro-fibrotic cytokines, which is the primary regulator of collagen synthesis in human skin [7] and normally exerts tumor-suppressive effects by enforcing cytostasis and differentiation. TGF-β must first combine with its specific receptor complex, including TGF-β type I (TβRI) and TGF-β type II (TβRII) receptor chains on the cell surface, to exert its biological effects [8,9]. However, as the tumor advances in malignant progression, the genome often accumulates mutations in the *TGF-β receptor* genes that renders the cancer cells unresponsive to TGF-β [10]. Moreover, its multiple effects on non-malignant stromal cells, such as evasion of immune surveillance, can be exploited by the tumor and hence turn TGF-β into a tumor-promoting factor that leads to increased invasion and metastasis [11]. Increased expression of *TGF-β* and activation of immune response suppressor pathways characterize the peri-tumoral skin microenvironment both in non-melanocytic cancers and in melanoma [12,13].

It is well known that SNPs in *TGF-β1*, *TGF-β2*, *TGF-βR1* and *TGF-βR2* genes are susceptibility factors for different type of cancers [14]. Less it is known about the role that polymorphisms of the *TGF-β* gene family may have in skin cancer, in particular in NMSC. The polymorphism at position +915 in the signal sequence (*rs1800471*), which changes codon 25 (arginine/proline), is associated with inter-individual variation in levels of TGF-β1 production. The intronic *TGF-β2 rs900* has no predicted effects in folding, activity or function of the TGF-β2 protein. On the other hand, T allele might interfere with ribosomal translation and rate of protein production [15].

*TGF-βR1 rs334349* and *rs334348* are in strict linkage disequilibrium. These SNPs are implied in altered miRNA interaction and differential germline allele specific expression of TGF-β Receptor I. *TGF-βR2 rs4522809*, situated in intron 2 of the *TGF-βR2*, seems not to affect mRNA folding and does not seem related to either TGF-β or TGF-βR2 protein expression [16]; on the other hand, there is the possibility that it is a marker in linkage with another causal variant [17].

One of the central mechanisms in cancer progression and expansion is the formation of new blood vessels from pre-existing vasculature. New angiogenesis actually plays a key role in tumor development, invasion, and metastases formation of many cancers, including NMSC [18,19]. Vascular Endothelial Growth Factor (VEGF) family is considered to be a fundamental key factor involved in angiogenesis, influencing progression, prognosis and therapy in various forms of cancers [20,21,22,23,24,25,26]. Specific variations of *VEGF* genes have been demonstrated to be genetic determinants for susceptibility, outcome and therapy response, especially for the solid tumors. *VEGF-A rs3025039* (*+936 C/T*) is located in the 3′ untranslated region of the gene. At a biological level, the T allele has been observed to give rise to lower circulating VEGF-A levels due to a loss of a potential binding site for the transcription factor activator protein-4 which may lower VEGF-A production [27,28].

Considering the strict functional interplay between TGF-β and VEGF pathways both in normal and neoplastic tissues [29] we have evaluated the role of some functionally relevant SNPs of the *TGF-β* gene family and of *VEGF-A* as susceptibility markers for NMSC.

## 2. Materials and Methods

### 2.1. Patient and Control Populations

Our study included 70 patients born in Western Sicily who were enrolled at time of their admission to the Dermatology Unit of Palermo, “Paolo Giaccone” University Hospital. Patients were affected by non-melanoma skin cancer, clinically and histologically classified as basal cell carcinomas or squamous cell carcinomas. To perform comparative genotype analyses 161 age related healthy controls were also enrolled. Demographic and clinical characteristics of patients and control subjects are summarized in Table 1. In addition, both patients and controls belonged to same ethnic group, since their parents and grandparents were born in Western Sicily.

Our study was performed in accordance with ethical standards of the Helsinki Declaration of the World Medical Association and Italian legislation, and was approved by the local institutional review board (Comitato Etico Palermo 1, protocol code CET1 03/2017, date of approval 1 March 2017). All participants gave their informed consent. Data were encoded to ensure privacy protection of patients and controls. Laboratory procedures were performed by lab staff unaware of the patient or control sources of the biological specimens.

### 2.2. Molecular Typing

As reported in Table 2, we selected six functional and common SNPs of the *TGF-β* pathway and one of the *VEGF-A* gene. Selection of these SNPs was performed using information acquired from dbSNP NCBI, the ENSEMBL database (http://www.ensembl.org/index.html, accessed on 3 April 2022), and the UCSC Genome Browser website (http://genome.ucsc.edu, accessed on 3 April 2022). Alleles and genotypes were analyzed using on demand assays developed by KBioscience Ltd. (Middlesex, UK). Tests apply homogeneous Fluorescence Resonance Energy Transfer (FRET) detection and allele specific PCR (Kaspar) as previously described [30]. The genotypes were determined using the 7300 system SDS software, vs. 1.3 (Applied Biosystems, Monza (Mi) Italy) sample by sample, on the basis of the detection of unique (homozygous samples) or double (heterozygous samples) fluorescence signals.

### 2.3. Statistical Analysis

Allele and genotype frequencies were evaluated by gene count using an online statistical analysis tool applied to the evaluation of SNPs (https://www.snpstats.net/start.htm, accessed on 9 May 2022). Hardy–Weinberg equilibrium was checked by Pearson’s test. Evaluation of power of calculation was performed with an online tool (https://sample-size.net/, accessed on 19 April 2022). Significant differences in genotype distributions between groups were calculated using chi-square or Fisher’s exact test applied to the appropriate 2 × 2 or 3 × 2 contingency tables. Overdominant, codominant, dominant and recessive models of heredity were applied in multiple logistic regression analyses adjusting the results for age and gender. GraphPadInStat software (GraphPad, San Diego, CA, USA, version 3.06) and the above mentioned online statistical analysis tool applied to the evaluation of the association of SNPs with diseases in the presence of other variances (biological, genetic, or clinical), were used to calculate statistical significance (*p*-value cutoff < 0.05) of odds ratio (OR) values and 95% confidence intervals (95% CI). Epistatic interaction among the different SNPs was performed using the Haplotype frequencies estimation (frequency threshold for rare haplotype: 0.01) and Haplotype association with response estimation functions at https://www.snpstats.net/start.htm (accessed on 19 April 2022) searching for so called pseudo-haplotypes. Actually, the concept of epistasis analysis is very similar in theory to haplotype analysis [33,34], in particular when functionally well-defined pathways (i.e., TGF-β and VEGF functionally interacting pathways) are studied.

## 3. Results

### 3.1. Allele and Genotype Frequencies of TGF-β and VEGF-A SNP in Non Melanocitic Skin Cancer Patient (NMSC) and Control Subjects

No significant differences were observed analyzing allelic and genotypic frequencies of *TGF-β1*, *TGF-β2*, and *TGF-βR1* gene SNPs between NMSC patient and control subjects. When *TGF-βR2 rs1800629* SNP was analyzed, significantly decreased frequencies of G allele and related genotypes were observed in the patient group (crude analysis) (Table 3) These results were confirmed applying the dominant model of heredity in multiple logistic regression analyses, adjusting the results for age and gender (Table 3). In addition, both *VEGF-A rs3025039T* allele and TT homozygous genotype were found to be significantly increased in patients compared to the controls both in the crude analyses and when the recessive model of heredity in multiple logistic regression analyses was applied, adjusting the results for age and gender (Table 3).

### 3.2. SNP Frequencies of NMSC Patient and Control Groups Stratified According to 64 Years Age Cut Off and Gender

As reported in Table 1, the median age of the NMSC patients was 64 years old. This median value was chosen as cut-off for breakdown of the two patient and control populations in subgroups of <64 and ≥64 year old subjects. Data reported in Table 4 showed that by analyzing SNP frequencies stratified according to age cut off and adjusted by gender, there was an increase in TGF-βR2 rs4522809A homozygous genotype frequency in patients compared to the controls both in the <64 and ≥64 year old classes whereas heterozygous genotype and frequency of G positive genotypes on the whole were decreased.

On the other hand, when the analysis was applied to VEGF-A rs3025039 genotypes, significant differences were observed only between ≥64 years old patients and controls. In particular VEGF-A rs3025039CC homozygous genotype frequency was significantly decreased with respect to the control group, whereas heterozygous genotype and frequency of T positive genotypes on the whole was significantly increased. In addition, homozygous T genotype reached roughly a statistical significance when considered separately.

Data reported in Table 5 show that *TGF-βR2 rs4522809A* homozygous genotype frequency was significantly increased in patients respect to the controls both in females and in males. In addition, the frequency of G positive genotypes, on the whole, was significantly decreased (homozygous + heterozygous genotypes). Decreases of both homozygous and heterozygous G genotypes, considered separately, reached roughly statistical significance.

On the other hand, when the analysis was applied to *VEGF-A rs3025039* genotypes no significant differences were observed stratifying data according to gender, even if the frequency of *rs3025039T* homozygous genotype was increased with a statistical significancy near to *p* = 0.05 threshold (0.083 and 0.068, respectively, for female and male groups). The complete analyses of data stratified according to age cut-off and gender for all SNP types are reported in Supplementary Tables S1 and S2.

### 3.3. SNP Frequencies in NMSC Patients Stratified according to Skin Cancer Hystotype

SNP frequencies were analyzed stratifying patient data according to NMSC hystotype as affected by basal cell carcinoma (BCC) and as affected by squamous cell carcinoma (SCC) (adjusted by 64 years age cut off and gender), and compared with each other and with the control population (Table 6). This would mean that 20 patients were represented in the SCC and 50 in the BCC group. However, the analyses confirmed that *TGF-β* R2 rs4522809G positive genotype frequencies are reduced in NMSC patients with respect to the controls (BCC dominant model OR 0.24, CI 95% 0.12–0.49, *p* < 0.0001; SCC dominant model OR 0.15, CI 95% 0.05–0.45, *p* = 0.0002) irrespective of BCC or SCC hystotype. Similarly, *VEGF-A rs3025039T* positive genotypes are significantly increased with respect to the controls both in BCC (recessive model OR 3.94, CI 95% 1.15–13.49, *p* < 0.0001) and SCC patients (dominant model OR 2.86, CI 95% 1.09–7.50, *p* = 0.034). On the other hand, the analysis of *TGF-βR1* SNP genotype frequencies showed that *rs334349AA* genotype is significantly increased in SCC patients compared both to the BCC (recessive model OR 6.20, CI 95% 1.25–30.81, *p* = 0.022) and control group (recessive model OR 4.31, CI95% 1.32–14.08, *p* = 0.022). The complete analyses of data stratified according to skin cancer hystotype for all the six SNP types are reported in Supplementary Table S3.

### 3.4. TGF-β and VEGF-A SNP Pseudo-Haplotype Associations with Non-Melanocytic Skin Cancer

Algorithms for haplotype frequencies estimation and association with the disease were used to evaluate the epistatic effect of combinations of genotypes at multiple loci on a complex trait such as NMSC due to the so-called gametic phase disequilibrium, as the alleles non-casually segregate within gametes, but are not physically located on the same chromosome [35]. This approach allows us to identify 31 functional pseudo-haplotypes composed by functional disequilibrium among alleles of functionally linked loci with a frequency > 0.01 in at least one of the populations analyzed, i.e., the NMSC or control population (Supplementary Table S4).

As reported in Table 7, pseudo-haplotype 2 composed by major alleles of *TGF-β1*, *TGF-β2*, *TGF-βR1* and *VEGF-A* SNPs and minor allele of *TGF-βR2* SNP showed a significantly reduced frequency in NMSC with respect to the control population.

On the contrary, pseudo-haplotype 14, composed by major alleles of *TGF-β1, TGF-β2*, *TGF-βR1* and *TGF-βR2* SNPs and minor allele of *VEGF-A* had a significantly increased frequency in NMSC patients. In addition, pseudo-haplotype 24 composed by minor alleles of *TGFβ2*, and both *TGFβR1* SNPs and major alleles of *TGF-β1*, *TGF-β2* and *VEGF-A* SNPs showed a significantly increased frequency in NMSC respect to control subjects. Complete pseudo-haplotype statistical analyses are reported in Supplementary Table S4.

Taken together, these data seem to indicate that TGF-β and VEGF-A pathway modulation by gene variants might have a role in susceptibility to or protection against NMSC.

## 4. Discussion

Non-melanoma skin cancers, basal cell and squamous cell carcinoma (BCC and SCC) commonly arise in the epidermis or hair follicles where proliferating basaloid tumor cells are present. Cytokine and growth factors are involved in this process [36].

Vascular endothelial growth factors (VEGFs) are the key regulators in angiogenesis and have been shown to play a significant role in the progression and prognosis of angiogenesis-related diseases, such as cancer [37,38,39,40]. VEGF is known to play a critical role in the development of non-melanoma skin cancers. Actually, VEGFs can affect skin carcinogenesis by directly interacting with keratinocytes, tumor cells, and immune cells [41,42]. In addition, VEGFs carry out other functions beyond their well-established effects on angiogenesis and influence skin carcinogenesis by directly activating tumor cells [19]. Moreover, several studies have uncovered direct effects of VEGF on keratinocytes and skin tumor cells. These studies suggested that in addition to enhancing angiogenesis, VEGF may promote skin carcinogenesis by altering the survival, proliferation, or stemness of keratinocytes and tumor cells in an autocrine manner [37,39,41,43].

Our data indicate that the frequency of *VEGF-A rs3025039T* positive genotypes was increased in NMSC patients, adjusting data for gender and age. Stratifying data according to age, T positive on the whole was increased in >64 years old subjects. Similarly, stratifying data according to gender, homozygous T genotype reached a roughly statistical significance when considered separately although this finding is dependent on a small number of subjects categorized according to gender and single genotype.

As above reported, the *VEGF-A rs3025039*T allele has been observed to give rise to lower circulating VEGF levels. However, studies on a South Italy population have demonstrated that the *rs3025039C/T* associated VEGF levels are variable and influenced by the presence of other *VEGF-A* SNP alleles in linkage disequilibrium with *rs3025039C/T* alleles [44]. A possible link between the *rs3025039C/T* SNP and several cancer types has been reported, and the contribution of this polymorphism to oncogenesis has been investigated in several types of tumors [45,46,47,48]. In some cases, T allele was found associated to protection against cancer [27,49]. Allele T associated susceptibility has been reported for oral cancer [50], head and neck cancer [51], breast cancer [52] and colorectal cancer [53,54]. In a recent paper, Kämmerer et al. [55], reporting the association of T allele with oral squamous cell carcinoma, showed that *VEGF-A rs3025039T* allele seems to be associated with an increased CD31 immuno-stain, currently used for measurement of microvessel density (MVD) and endothelial cell kinetic in normal and pathological tissues.

On the other hand, our data indicated that the statistical significance of the increase in the T positive genotype was maintained both in patients ≥64 years old and in patients affected by BCC. Recent findings suggest that in aging the reduced angiogenesis potential correlates with a reduced expression of *VEGF*-A [56]. Moreover, it appears intriguing that tumor cells of human BCCs tend to show weak VEGF expression with positive tumor cells predominantly localized to the invading margin [26,57]. In contrast, SCCs, which are typically more aggressive than BCCs, display more intense and widespread staining, with higher expression in tumor cells localized near infiltrating inflammatory cells [26]. However, our group of SCC patients is small as only 20 patients were represented in the SCC group, so the data obtained by comparing SCC patients with BCC and controls should be considered suggestive but not conclusive. In addition, the case mixes in both BCC and SCC groups (Table 1) do not allow us to further analyze possible significant differences associated with histotype, therapeutical approach, or relapses in relationship with SNP types.

Overall, the increased frequency of *VEGF-A rs3025039T* positive genotypes in our group of patients confirms the view that analysis of *VEGFs* genetic polymorphisms in aged cancer patients might be important for a better tailoring of cancer therapy [56].

The TGF-β signaling pathway plays an important role in the regulation of normal and cancer skin cell proliferation [13]. Increased expression of TGF-β and activation of the T regulatory pathway characterize the peri-tumoral skin microenvironment both in non-melanocytic tumors and in melanoma. TGF-β1 can modulate normal and pathological cell differentiation and proliferation in an auto- or paracrine manner [58]. In particular, the ability of TGF-β signaling to activate target genes enables the pathway to impact diverse cellular processes including proliferation, differentiation, migration, apoptosis, and endothelial cell remodeling [59,60] with different and sometimes opposite effects on cancer cells. Actually, TGF-β normally exerts tumor-suppressive effects. However, as the tumor advances in malignant progression, mutations in the TGF-β receptor system render the cancer cells unresponsive to the cytokine and to TGF-β through its multiple effects on non-malignant stromal cells, such as evasion of immune surveillance, creating a tumor-promoting factor that leads to increased invasion and metastasis [11].

In this scenario, given the role of genetic polymorphisms located in both transcribed and untranscribed regions of *TGF-β* and *TGF-β* receptors, the tuning of TGF-β effects might be crucial.

Accordingly, we found that *TGF-βR2* SNP genotypes are differently distributed in NMSC patients and controls. In particular, *TGF-βR2 rs4522809G* positive genotype frequencies are reduced in NMSC patients with respect to the controls, adjusting data for gender and age. Similar results were obtained by analyzing genetic frequencies in female, male, younger or older patients compared to adequate controls. Moreover, this reduced frequency was observed both in SCC and BCC histologic types. Decreases of both homozygous and heterozygous G genotype reach roughly statistical significance when considered separately but this finding is dependent on a relatively small number of subjects categorized according to gender and single genotype.

It is not yet clear how TGF-β signaling is influenced by *rs4522809*. It has been hypothesized that the G allele of *TGF-βR2 rs4522809*, that is located in the intronic region, might modify the gene expression. Alternatively, it is possible that this gene variant regulates the amount and direction of transcription [61]. In this view, the increased frequency of *rs4522809G* positive genotypes in the control group allows to speculate about a possible protective effect of this gene variant against NMSC. It appears intriguing that a similar protective effect of the G allele was found in other types of cancer such as breast cancer [17]. We have also found that *TGF-βR1 rs334349AA* genotype frequencies are significantly increased in SCC patients compared both to BCC and controls. Association of this *TGF-βR1* variant with cancer was documented in particular for colon rectal cancer [62]. Overall, our results, even if they need to be confirmed in a larger sample of NMSC patients (in particular for data comparing SCC and BCC histologic types) suggest that the net effect of the *TGF-β* gene pathway on skin cancer pathogenesis is influenced by polymorphisms of genes coding for the TGF-β receptor complex.

As is well known, the net effect of the presence of a functionally relevant SNP might be masked or counterbalanced by other SNPs in the same or in interacting genes [31]. Our data in their complexity seem to suggest both protective and susceptible polymorphism in strictly interacting genes as *TGF-β* family and *VEGF-A* [63,64]. In this regard, we have explored the interaction among the six SNP types, applying an online haplotype searching tool to determine the so called functional pseudo-haplotypes [32]. This would describe the correlation among genotypes of loci not lying on the same chromosome, that has been also described as “gametic phase disequilibrium”, i.e., the non-random association of alleles within gametes, even for physically unlinked loci tethered on different chromosomes [65]. This approach allowed us to establish that two pseudo-haplotypes showed a significantly different distribution frequency in NMSC with respect to the control population, implicating a minor allele of *TGF-βR2* or minor allele of *VEGF-A* SNPs combined with major alleles of the other SNPs studied. Analyzing the frequencies of the two *TGF-βR2* and *VEGF-A* SNPs individually, it seems that the first haplotype is associated with a protective effect against NMSC and the second with susceptibility. These findings would suggest that the effect of *TGF-βR2* and *VEGF-A* SNPs might be more evident when the major alleles of the other loci are present. It is intriguing to observe that when minor alleles of *TGF-βR2* and *VEGF-A* SNPs are contemporaneously associated in the same pseudo-haplotype, no significant differences between frequencies of NMSC and control were observed (Table S4 pseudo-haplotypes 12 and 16). Finally, pseudo-haplotype analyses revealed that the pseudo-haplotype 24 composed by minor alleles of *TGF-β2* rs900, *TGF-βR1 rs334348* and *rs4522809* SNPs and major alleles of *TGF-β1*, *TGF-βR2* and *VEGF-A* SNPs might be a susceptibility factor for NMSC.

On the other hand, both next generation sequencing studies and GWAS have not directly evidenced that SNP of *TGF-β* pathway or *VEGF-A* are genetic markers of risk for NMSC. These approaches have highlighted that pigmentation genes as *TYR*, *TYRP1*, *OCA2*, *SLC24A5*, *SLC45A2*, *POMC*, *ASIP*, *ATRN*, involved in activation of the Hedgehog (HH) pathway, were identified as susceptible loci for BCC and SCC, independently of the pigmentation phenotypes [66,67]. *HLA class II*, *TP63, FOXP1* genes were associated with NMSC [68,69]. However, novel approaches that integrate skin expression-related single-nucleotide polymorphisms (eSNPs) and pathway analysis based on Kyoto Encyclopedia of Genes and Genomes (KEGG), Gene Ontology (GO), or BioCarta databases allowed identification of the role of genes of the *TGF-β* pathway interacting with genetic activation of the Hedgehog pathway [70,71]. The NO pathway in the BioCarta includes VEGFA SNPs and plays an important role in the regulation of vascular endothelial function facilitating, in particular, BCC development and an angiogenic response propagated by the growing skin cancer [72]. In this view, it is hypothesized that *VEGF-A* and *TGF-βR2* genetic variants studied by our group can be considered components of multiloci and complex metabolic pathways that characterize NMSC development.

## 5. Conclusions

In conclusion, our data suggest that a complex interplay among the genetic polymorphisms of *TGF-β*, *TGF-β* receptors and *VEGF-A* genes might influence the net effect of the genetic background of the patients on development of non-melanocytic tumors of the skin. Actually, in humans, their gene products can mediate stimulating or inhibiting cell growth effects depending on the cellular and tissue targets [10]. TGF-β, in particular, might mediate different and time-differentiated effects on tumor evolution. The apparent contrasting effects might be due to progressively distinct genetic states and modifications of the tumor microenvironment influencing proliferation, metabolism and expression of key genes targeted [34,58,73]. In this view, larger investigations are warranted to explore the relevance of the TGF-β and VEGF pathway genetic background as a diagnostic or predictive marker that can be targeted to treat non-melanocytic skin cancers.

## Figures and Tables

**Table 1:** Demographic and clinical characteristics of 70 patients with non-melanocytic skin cancer (NMSC) and 161 control subjects.

Demographic Characteristics	Cancer			Controls		*p*
	N.		%	N.	%	
Age, mean ± SD	63.90 ± 14.38			50.37 ± 11.49		<0.0001
Age median	64.00			46.00		
≥64 years old	36		51.42	48	29.81	-
Women	24		34.33	76	47.81	0.0719
Basal cell carcinoma (BCC)	50		71.42	-	-	-
Squamous cell carcinoma (SCC)	20		28.58	-	-	-
Non-melanocytic skin cancer patient clinical characteristics						
BCC Subtypes:	N.	%	Metastases		Therapy	
Nodular	33	66.00	No		Surgical excision	
Superficial	12	24.00	No		Surgical excision	
Adenoid BCC	2	4.00	No		Surgical excision	
Nodulocystic BCC	1	2.00	No		Surgical excision	
Micronodular BCC	1	2.00	No		Mohs surgery	
Pinkus Fibroepithelioma	1	2.00	No		Surgical excision	
SCC Subtypes:						
SCC arising in actinic keratosis	14	70.00	No		Surgical excision	
Keratoacanthoma	2	10.00	No		Immune stimulatory therapy and cryosurgery	
Adenosquamous carcinoma	1	5.00	No		Surgical excision and adjuvant immunotherapy	
Verrucous SCC	1	5.00	No		Surgical excision	
Invasive Bowen’s disease	1	5.00	N.D.		Cryosurgery and adjuvant chemotherapy	
Carcinosarcoma	1	5.00	Yes		Surgical excision and adjuvant chemotherapy	

**Table 2:** Genes, reference number (rs), localization and position of SNPs investigated in the study.

Genes	SNPs	Gene Region	Position	Major Allele	Minor Allele	MAF *	Biological Effect	Reference
*TGF-β1*	*rs1800471*	Exon 1	19:41352971	G	C	0.05	Modification of TGF-β1 production	[31]
*TGF- β* *2*	*rs900*	3′UTR	1:218441563	A	T	0.29	Modification of TGF-β2 production	[15]
*TGF-β* *R1*	*rs334348*	3′UTR	9:99150189	A	G	0.26		[32]
	*rs334349*	3′UTR	9:99152105	G	A	0.27		
*TGF-β* *R2*	*rs4522809*	Intron 2	3:30627192	A	G	0.47	Probable association to TGF-β signaling modification	[17]
*VEGF-A*	*rs3025039*	3′UTR	6:43784799	C	T	0.14	Reduced VEGF-A production	[27,28]

* Minor allele frequencies (MAF) in Caucasian population retrieved from dbSNP database (https://www.ncbi.nlm.nih.gov/snp/, accessed on 5 June 2022).

**Table 3:** Significantly different allelic and genotypic SNP frequencies in 70 non-melanocitic skin cancer patients (NMSC) and 161 control subjects.

GENE	SNP	Alleles/Genotypes	Controls		NMSC		OR (95% CI)	*p*-Value
			Nr	Freq.	Nr	Freq.		
*TGF-β1*	*rs1800471*	G	292	0.91	123	0.88		0.774
		C	30	0.09	17	0.12		
		G/G	135	0.84	56	0.8	1.27 (0.72–2.26)	
		C/G	22	0.14	11	0.16		0.757
		C/C	4	0.02	3	0.04		
*TGF-β2*	*rs900*	A	222	0.69	94	0.67		0.822
		T	100	0.31	46	0.33		
		A/A	75	0.47	34	0.49	1.08 (0.71–1.65)	
		A/T	72	0.45	26	0.37		0.673
		T/T	14	0.09	10	0.14		
*TGF-βR1*	*rs334348*	A	244	0.76	106	0.76		1.000
		G	78	0.24	34	0.24	0.97 (0.52–1.79)	
		A/A	96	0.6	42	0.6		0.990
		A/G	52	0.32	22	0.31		
		G/G	13	0.08	6	0.09		
	*rs334349*	G	250	0.78	99	0.71		0.845
		A	72	0.22	41	0.29		
		G/G	101	0.63	37	0.53	1.42 (0.77–2.62)	
		G/A	48	0.3	25	0.36		0.343
		A/A	12	0.07	8	0.11		
* *TGF-βR2*	*rs4522809*	A	188	0.58	110	0.79		<0.0001
		G	134	0.42	30	0.21		
		A/A	55	0.34	48	0.69	0.21 (0.10–0.41)	
		A/G	78	0.48	14	0.2		<0.0001
		G/G	28	0.17	8	0.11		
** *VEGF-A*	*rs3025039*	C	272	0.84	104	0.74		0.0291
		T	50	0.16	36	0.26		
		C/C	116	0.72	43	0.61	1.86 (1.14–2.34)	
		C/T	40	0.25	18	0.26		0.022
		T/T	5	0.03	9	0.13		

* Dominant model (A/A vs. A/G-G/G) OR (95% C.I.) = 0.24 (0.13–0.43) *p* < 0.0001. ** Recessive model (T/T vs. C/C-C/T) OR (95% C.I.) = 4.60 (1.48–14.29) *p* < 0.0067.

**Table 4:** Significantly different *TGF-βR2* and *VEGF-A* SNP frequencies in non melanocitic skin cancer patients (NMSC) and controls (CTRL) stratified according to 64 years age cut off (adjusted by gender).

Genes and SNP Alleles		Age < 64				Age ≥ 64			
		CTRL	NMSC	OR (95% CI)	*p*-Value	CTRL	NMSC	OR (95% CI)	*p*-Value
		N (Gen. Freq.)				N (Gen. Freq.)			
*TGF-βR2* *rs4522809*	A/A	42 (0.37)	23 (0.68)	3.54 (1.57–7.98)	0.0028	13 (0.27)	25 (0.69)	6.12 (2.36–15.9)	0.0002
	A/G	54 (0.48)	7 (0.20)	0.28 (011–0.71)	0.0053	24 (0.50)	7 (0.20)	0.15 (0.05–0.45)	0.0058
	G/G	17 (0.15)	4 (0.12)	1.22 (0.75–2.41)	0.784	11 (0.23)	4 (0.11)	0.42 (0.12–1.45)	0.249
	G/*	71 (0.63)	11 (0.32)	0.28 (0.12–0.64)	0.0028	35 (0.73)	11 (0.31)	0.16 (0.06–0.42)	0.0002
*VEGF-A* *rs3025039*	C/C	77 (0.68)	25 (0.74)	1.29 (0.55–3.06)	0.673	39 (0.81)	18 (0.50)	0.23 (0.09–0.61)	0.0042
	C/T	33 (0.29)	6 (0.17)	0.52 (0.19–1.37)	0.267	7 (0.15)	12 (0.33)	3.74 (1.23–11.33)	0.0058
	T/T	3 (0.03)	3 (0.09)	3.55 (0.70–18.5)	0.137	2 (0.04)	6 (0.17)	4.59 (0.87–24.33)	0.0632
	T/*	36 (0.32)	9 (0.26)	0.77 (0.33–1.82)	0.673	9 (0.19)	18 (0.50)	4.33 (1.63–11.5)	0.0042

G/* = G positive genotypes of *TGF-βR2 rs4522809*A/G SNP; T/* = T positive genotypes of *VEGF-A rs3025039*C/T SNP.

**Table 5:** Significantly different SNP frequencies in non melanocitic skin cancer patients (NMSC) and controls (CTRL), stratified according to gender (adjusted by age cut off).

Genes and SNP Alleles		Female				Male			
		CTRL	MNSC	OR (95% CI)	*p*-Value	CTRL	MNSC	OR (95% CI)	*p*-Value
		N (Gen. Freq.)				N (Gen. Freq.)			
*TGF-βR2* *rs4522809*	A/A	31 (0.40)	17 (0.71)	3.68 (1.37–9.91)	0.011	24 (0.29)	31 (0.67)	5.08 (2.33–11.1)	0.0001
	A/G	37 (0.47)	4 (0.17)	0.22 (0.07–0.71)	0.008	41 (0.49)	10 (0.22)	0.28 (0.13–0.65)	0.003
	G/G	10 (0.13)	3 (0.12)	0.97 (0.24–3.86)	1.000	18 (0.22)	5 (0.11)	0.44 (0.15–1.28)	0.153
	G/*	47 (0.60)	7 (0.29)	0.27 (0.10–0.73)	0.011	59 (0.71)	15 (0.33)	0.20 (0.09–0.43)	0.0001
*VEGF-A* *rs3025039*	C/C	54 (0.69)	13 (0.54)	0.53 (0.21–1.34)	0.221	62 (0.75)	30 (0.65)	0.64 (0.29–1.39)	0.311
	C/T	22 (0.28)	8 (0.33)	1.27 (0.48–3.39)	0.618	18 (0.21)	10 (0.22)	1.03 (0.42–2.40)	1.00
	T/T	2 (0.03)	3 (0.13)	5.43 (0.85–34.7)	0.083	3 (0.04)	6 (0.13)	4.08 (0.95–16.8)	0.068
	T/*	24 (0.31)	11 (0.46)	1.90 (0.75–4.86)	0.221	21 (0.25)	16 (0.35)	1.57 (0.72–3.45)	0.311

**Table 6:** Significantly different SNP frequencies in non melanocitic skin cancer patients (NMSC) affected by basal cell carcinoma (BCC) compared to NMSC with squamous cell carcinoma (SCC) (adjusted by 64 years age cut off and gender).

Genes and SNP Alleles		BCC		SCC		Controls		BCC vs. SCC		BCC vs. Controls		SCC vs. Controls	
		Nr	Freq.	Nr	Freq.	Nr	Freq.	OR 95% CI	*p* Value	OR (95% CI)	*p* Value	OR (95% CI)	*p* Value
* *TGF-βR1* *rs334349*	G/G	29	0.58	8	0.40	101	0.63	0.48 0.17–1.39	0.158	0.82 0.43–1.57	0.618	0.39 0.15–1.02	0.057
	G/A	18	0.36	7	0.35	48	0.3	1.39 0.41–4.71	0.882	1.37 0.68–2.78	0.61	1.84 0.63–5.42	0.615
	A/A	3	0.06	5	0.25	12	0.07	7.09 1.31–38.48	0.019	0.81 0.21–3.18	1.000	5.48 1.52–19.7	0.044
** *TGF-βR2* *rs4522809*	A/A	33	0.66	15	0.75	55	0.34	1.54 0.48–4.98	0.444	3.74 1.91–7.31	0.0001	5.78 1.99–16.7	0.001
	A/G	12	0.24	2	0.10	78	0.48	0.35 0.07–1.81	0.145	0.24 0.11–0.52	0.0002	0.09 0.02–0.40	0.0004
	G/G	5	0.10	3	0.03	28	0.17	1.31 0.27–6.35	0.567	0.53 0.19–1.45	0.268	0.84 0.23–3.06	1.000
*** *VEGF-A* *rs3025039*	C/C	33	0.66	10	0.50	116	0.72	0.52 0.86–8.27	0.279	0.75 0.38–1.48	0.478	0.39 0.15–1.00	0.068
	C/T	10	0.20	8	0.40	40	0.25	2.67 1.00–13.00	0.129	0.99 0.44–2.23	0.091	2.59 0.94–7.17	0.085
	T/T	7	0.14	2	0.10	5	0.03	1.20 0.20–7.13	0.745	5.08 1.53–16.8	0.0085	4.89 0.82–29.1	0.074

* *rs334349*: SCC vs. BCC recessive model 6.20 (1.25–30.81) *p* = 0.022; SCC vs. Controls recessive model 4.31 (1.32–14.08) *p* = 0.024. ** *rs4522809*: BCC vs. Controls dominant model 0.24 (0.12–0.49) *p* < 0.0001; SCC vs. Controls dominant model 0.15 (0.05–0.45) *p* = 0.0002. *** *rs3025039*: BCC vs. Controls recessive model 3.94 (1.15–13.49) *p* < 0.0001; SCC vs. Controls dominant model 2.86 (1.09–7.50) *p* = 0.034.

**Table 7:** Significantly different results obtained analyzing frequencies of 31 pseudo-haplotype (p-Hp) containing major or minor alleles of *TGF-β* and *VEGF-A* SNPs, in non-melanocytic skin cancer (NMSC) patients and control (CTRL) subjects.

p-Hp	*TGF-β* *1 rs1800471 G/C*	*TGF-β2* *rs900* *A/T*	*TGF-β* *R1* *rs334348 A/G*	*TGF-β* *R1 rs334349 G/A*	*TGF-β* *R2* *rs4522809* *A/G*	*VEGF-A* *rs3025039* *C/T*	CTRL	NMSC	OR (95%CI)	*p* Value
2	G	A	A	G	G	C	0.150	0.028	0.17 (0.04–0.73)	0.0061
14	G	A	A	G	A	T	0.010	0.124	11.7 (2.46–55.9)	0.0005
24	G	T	G	A	A	C	0.000	0.058	21.8 (1.16–411)	0.0079

## Data Availability

All data generated or analyzed during this study are stored in electronic archives and can be supplied on request.

## Supplementary Materials

The following supporting information can be downloaded at: https://www.mdpi.com/article/10.3390/genes13071235/s1, Table S1: SNP frequencies in Non Melanocitic Skin Cancer Patients (NMSC) and Controls (CTRL) stratified according to 64 years age cut off (adjusted by gender); Table S2: SNP frequencies in Non Melanocitic Skin Cancer Patients (NMSC) and Controls (CTRL) stratified according to gender (adjusted by age cut off); Table S3: SNP frequencies in Non Melanocitic Skin Cancer Patients (NMSC) affected by basal cell carcinoma (BCC) compared to NMSC with squamous cell carcinoma (SCC) (adjusted by 64 years age cut off and gender); Table S4: Analysis of frequencies of 31 pseudo-haplotype (p-Hp) containing major or minor alleles of *TGF-β* gene family and *VEGF-A* SNPs, in non-melanocytic skin cancer (NMSC) patients and controls (CTRL).
