# Supplementary material for: TGF-β/VEGF-A Genetic Variants Interplay in Genetic Susceptibility to Non-Melanocytic Skin Cancer
Source: Genes (Basel). 2022 Jul 13;13(7):1235. doi: 10.3390/genes13071235 (PMC9317818; doi:10.3390/genes13071235)
Supplement: Supplementary file 1 [file genes-13-01235-s001.zip › genes-1756596-supplementary.pdf]

**Table S1.** SNP frequencies in Non Melanocitic Skin Cancer Patients (NMSC) and Controls (CTRL) stratified according to 64 years Age cut off (adjusted by Gender)

| Genes and SNP alleles | | Age <64 | | | | Age ≥64 | | | |
|---|---|---|---|---|---|---|---|---|---|
| | | CTRL | NMSC | OR | *p*-value | CTRL | NMSC | OR | *p*-value: |
| | | N (gen. freq.) | | (95% CI) | | N (gen. freq.) | | (95% CI) | |
| *TGF-β1 rs1800471* | G/G | 95 (0.84) | 26 (0.76) | 1.00 | 0..32 | 40 (0.83) | 30 (0.83) | 0.93 (0.29-3.08) | 0.931 |
| | C/G | 16 (0.14) | 7 (0.21) | 1.60 (0.59-4.30) | 0.38 | 6 (0.13) | 4 (0.11) | 0.84 (0.22-3.26) | 0.797 |
| | C/C | 2 (0.02) | 1 (0.03) | 1.83 (0.16-20.9) | 0.68 | 2 (0.4) | 2 (0.06) | 1.27 (0.17-9.65) | 0.823 |
| | C/* | 18 (0.16) | 8 (0.24) | 1.62 (0.63-4.15) | 0.32 | 8 (0.17) | 35 (0.17) | 1.00 (0.31-3.19) | 0.931 |
| *TGF- β2 rs900* | A/A | 58 (0.51) | 15 (0.44) | 1.29 (0.59-2.83) | 0.557 | 17 (0.35) | 19 (0.53) | 2.04 (0.84-4.93) | 0.125 |
| | A/T | 46 (0.41) | 12 (0.35) | 0.82 (0.37-1.85) | 0.643 | 26 (0.54) | 14 (0.39) | 0.48 (0.19-1.30) | 0.191 |
| | T/T | 9 (0.08) | 7 (0.21) | 2.57 (0.86-7.69) | 0.100 | 5 (0.11) | 3 (0.08) | 0.52 (0.11-2.53) | 1.000 |
| | T/* | 53 (0.49) | 19 (0.56) | 0.76 (0.35-1.65) | 0.557 | 31 (0.65) | 17 (0.47) | 0.49 (0.20-1.19) | 0.125 |
| *TGF-β R1 rs334348* | A/A | 66 (0.58) | 20 (0.59) | 0.92 (0.42-2.03) | 0.999 | 30 (0.63) | 22 (0.61) | 0.94 (0.39-2.29) | 1.000 |
| | A/G | 37 (0.33) | 10 (0.29) | 0.85 (0.36-1.98) | 0.699 | 15 (0.31) | 12 (0.33) | 1.04 (0.40-2.71) | 0.993 |
| | G/G | 10 (0.09) | 4 (0.12) | 1.18 (0.34-4.14) | 0.801 | 3 (0.07) | 2 (0.06) | 0.86 (0.13-5.68) | 0.862 |
| | G/* | 47 (0.42) | 14 (0.41) | 3.30 (1.40-10.89) | 0.999 | 18 (0.38) | 14 (0.39) | 1.06 (0.44-2.58) | 1.000 |
| *TGF-β R1 rs334349* | G/G | 69 (0.61) | 18 (0.53) | 1.22 (0.55-2.70) | 0.430 | 32 (0.67) | 19 (0.53) | 0.56 (0.23-1.36) | 0.259 |
| | G/A | 35 (0.31) | 13 (0.38) | 1.24 (0.55-2.80) | 0.613 | 13 (0.27) | 12 (0.33) | 1.53 (0.58-4.04) | 0.566 |
| | A/A | 9 (0.08) | 3 (0.09) | 1.01 (0.25-4.04) | 0.992 | 3 (0.06) | 5 (0.14) | 2.73 (0.58-12.8) | 0.979 |
| | A/* | 44 (0.44) | 16 (0.72) | 0.71 (0.33-1.55) | 0.430 | 16 (0.33) | 17 (0.47) | 1.79 (0.74-4.35) | 0.259 |
| *TGF-β R2 rs4522809* | A/A | 42 (0.37) | 23 (0.68) | 3.54 (1.57-7.98) | 0.0028 | 13 (0.27) | 25 (0.69) | 6.12 (2.36-15.9) | 0.0002 |
| | A/G | 54 (0.48) | 7 (0.20) | 0.28 (011-0.71) | 0.0053 | 24 (0.50) | 7 (0.20) | 0.15 (0.05-0.45) | 0.0058 |
| | G/G | 17 (0.15) | 4 (0.12) | 1.22 (0.75-2.41) | 0.784 | 11 (0.23) | 4 (0.11) | 0.42 (0.12-1.45) | 0.249 |
| | G/* | 71 (0.63) | 11 (0.32) | 0.28 (0.12-0.64) | 0.0028 | 35 (0.73) | 11 (0.31) | 0.16 (0.06-0.42) | 0.0002 |
| *VEGF-A rs3025039* | C/C | 77 (0.68) | 25 (0.74) | 1.29 (0.55-3.06) | 0.673 | 39 (0.81) | 18 (0.50) | 0.23 (0.09-0.61) | 0.0042 |
| | C/T | 33 (0.29) | 6 (0.17) | 0.52 (0.19-1.37) | 0.267 | 7 (0.15) | 12 (0.33) | 3.74 (1.23-11.33) | 0.0058 |
| | T/T | 3 (0.03) | 3 (0.09) | 3.55 (0.70-18.5) | 0.137 | 2 (0.04) | 6 (0.17) | 4.59 (0.87-24.33) | 0.0632 |
| | T/* | 36 (0.32) | 9 (0.26) | 0.77 (0.33-1.82) | 0.673 | 9 (0.19) | 18 (0.50) | 4.33 (1.63-11.5) | 0.0042 |

**Table S2.** SNP frequencies in Non Melanocitic Skin Cancer Patients (NMSC) and Controls (CTRL) stratified according to Gender (adjusted by Age cut off)

| Genes and SNP alleles | | Female | | | | Male | | | |
|---|---|---|---|---|---|---|---|---|---|
| | | CTRL | MNSC | OR | *p*-value | CTRL | MNSC | OR | *p*-value: |
| | | N (gen. freq.) | | (95% CI) | | N (gen. freq.) | | (95% CI) | |
| *TGF-β1 rs1800471* | G/G | 62 (0.79) | 19 (0.79) | 0.88 (0.27-2.92) | 0. 876 | 73 (0.88) | 37 (0.81) | 1.84 (0.68-4.97) | 0. 211 |
| | C/G | 14 (0.18) | 4 (0.17) | 0.90 (0.25-3.27) | 0.889 | 8 (0.10) | 7 (0.15) | 1.76 (0.59-5.27) | 0.333 |
| | C/C | 2 (0.03) | 1 (0.04) | 0.80 (0.06-10.89) | 0.882 | 2 (0.02) | 2 (0.04) | 1.83 (0.25-13.61) | 0.565 |
| | C/* | 16 (0.21) | 5 (0.21) | 1.00 | 0.876 | 10 (0.12) | 9 (0.19) | 1.78 (0.66-4.75) | 0.211 |
| *TGF- β2 rs900* | A/A | 40 (0.51) | 11 (0.46) | 1.14 (0.43-2.99) | 0.821 | 35 (0.42) | 23 (0.50) | 2.19 (0.75-6.44) | 0.358 |
| | A/T | 32 (0.41) | 11 (0.46) | 1.19 (0.44-3.26) | 0.923 | 40 (0.48) | 15 (0.33) | 0.53 (0.24-1.20) | 0.114 |
| | T/T | 6 (0.8) | 2 (0.8) | 0.89 (0.14-5.62) | 0.788 | 8 (0.09) | 8 (0.17) | 1.66 (0.54-5.14) | 0.073 |
| | T/* | 38 (0.49) | 13 (0.54) | 0.81 (0.14-4.84) | 0.821 | 48 (0.57) | 35 (0.50) | 0.71 (0.34-1.48) | 0.358 |
| *TGF-β R1 rs334348* | A/A | 46 (0.59) | 14 (0.59) | 1.00 | 1.000 | 50 (0.60) | 28 (0.61) | 1.03 (0.49-2.19) | 1.000 |
| | A/G | 27 (0.35) | 8 (0.33) | 0.79 (0.28-2.28) | 0.739 | 25 (0.30) | 14 (0.30) | 1.04 (0.46-2.34) | 1.000 |
| | G/G | 5 (0.06) | 2 (0.08) | 1.11 (0.17-7.11) | 0.845 | 8 (0.10) | 4 (0.09) | 0.99 (0.27-3.74) | 0.949 |
| | G/* | 32 (0.41) | 10 (0.41) | 1.00 | 1.000 | 33 (0.40) | 18 (0.39) | 0.99 (0.47-1.98) | 1.000 |
| *TGF-β R1 rs334349* | G/G | 50 (0.64) | 15 (0.62) | 1.71 (0.34-8.56) | 0.783 | 51 (0.62) | 22 (0.48) | 0.57 (0.28-1.19) | 0.143 |
| | G/A | 23 (0.30) | 6 (0.25) | 0.72 (0.23-2.23) | 0.691 | 25 (0.30) | 19 (0.41) | 1.63 (0.77-3.46) | 0.246 |
| | A/A | 5 (0.06) | 3 (0.13) | 1.53 (0.29-8.03) | 0.527 | 7 (0.08) | 5 (0.11) | 1.32 (0.39-4.44) | 0.754 |
| | A/* | 50 (0.36) | 9 (0.38) | 0.87 (0.31-2.39) | 0.785 | 32 (0.38) | 24 (0.52) | 1.74 (0.84-3.60) | 0.143 |
| *TGF-β R2 rs4522809* | A/A | 31 (0.40) | 17 (0.71) | 3.68 (1.37-9.91) | 0.011 | 24 (0.29) | 31 (0.67) | 5.08 (2.33-11.1) | 0.0001 |
| | A/G | 37 (0.47) | 4 (0.17) | 0.22 (0.07-0.71) | 0.008 | 41 (0.49) | 10 (0.22) | 0.28 (0.13-0.65) | 0.003 |
| | G/G | 10 (0.13) | 3 (0.12) | 0.97 (0.24-3.86) | 1.000 | 18 (0.22) | 5 (0.11) | 0.44 (0.15-1.28) | 0.153 |
| | G/* | 47 (0.60) | 7 (0.29) | 0.27 (0.10-0.73) | 0.011 | 59 (0.71) | 15 (0.33) | 0.20 (0.09-0.43) | 0.0001 |
| *VEGF-A rs3025039* | C/C | 54 (0.69) | 13 (0.54) | 0.53 (0.21-1.34) | 0.221 | 62 (0.75) | 30 (0.65) | 0.64 (0.29-1.39) | 0.311 |
| | C/T | 22 (0.28) | 8 (0.33) | 1.27 (0.48-3.39) | 0.618 | 18 (0.21) | 10 (0.22) | 1.03 (0.42-2.40) | 1.00 |
| | T/T | 2 (0.03) | 3 (0.13) | 5.43 (0.85-34.7) | 0.083 | 3 (0.04) | 6 (0.13) | 4.08 (0.95-16.8) | 0.068 |
| | T/* | 24 (0.31) | 11 (0.46) | 1.90 (0.75-4.86) | 0.221 | 21 (0.25) | 16 (0.35) | 1.57 (0.72-3.45) | 0.311 |

C/*= C positive genotypes of *TGF-β1 rs1800471*G/C SNP; T/*= T positive genotypes of *TGF-β2 rs900*A/T SNP; G/*= G positive genotypes of *TGF-βR1 rs334348*A/G SNP; A/*= A positive genotypes of *TGF-βR1 rs334349*G/A SNP; G/*= G positive genotypes of *TGF-βR2 rs4522809*A/G SNP; T/*= T positive genotypes of *VEGF-A rs3025039*C/T SNP

**Table S3.** SNP frequencies in Non Melanocitic Skin Cancer Patients (NMSC) affected by basal cell carcinoma (BCC) compared to NMSC with squamous cell carcinoma (SCC) (adjusted by 64 years Age cut off and Gender)

| Genes and SNP alleles | | BCC | | SCC | | Controls | | BCC Vs SCC | | BCC Vs Controls | | SCC Vs Controls | |
|---|---|---|---|---|---|---|---|---|---|---|---|---|---|
| | | Nr | Freq. | Nr | Freq. | Nr | Freq. | OR 95% CI | *p* value | OR (95% CI) | *p* value | OR (95% CI) | *p* value |
| *TGF-β1 rs1800471* | G/G | 41 | 0.82 | 15 | 0.75 | 135 | 0.84 | 0.66 0.19-2.28 | 0.522 | 0.888 0.38-2.02 | 0.828 | 0.58 0.19- 1.73 | 0.346 |
| | C/G | 8 | 0.16 | 3 | 0.15 | 22 | 0.14 | 0.90 0.21-3.98 | 0.788 | 1.37 0.55-3.44 | 0.723 | 1.35 0.35-5.13 | 0.311 |
| | C/C | 1 | 0.02 | 2 | 0.10 | 4 | 0.02 | 6.35 0.51-79.12 | 0.133 | 0.63 0.07-6.15 | 1.000 | 4.54 0.75-27.6 | 0.133 |
| *TGF- β2 rs900* | A/A | 26 | 0.52 | 8 | 0.40 | 75 | 0.47 | 0.62 0.22-1.76 | 0.410 | 1.24 0.66-2.34 | 0.521 | 0.76 0.29-1.97 | 0.639 |
| | A/T | 18 | 0.36 | 8 | 0.40 | 72 | 0.45 | 1.43 0.45-4.59 | 0.711 | 0.63 0.31-1.29 | 0.38 | 0.95 0.33-2.72 | 0.362 |
| | T/T | 6 | 0.12 | 4 | 0.20 | 14 | 0.09 | 1.89 0.41-8.76 | 0.523 | 1.11 0.37-3.30 | 0.580 | 2.55 0.67-9.73 | 0.119 |
| *TGF-β R1 rs334348* | A/A | 29 | 0.58 | 13 | 0.65 | 96 | 0.6 | 0.48 0.17-1.39 | 0.559 | 0.93 0.49-1.78 | 0.870 | 1.26 0.48-3.32 | 0.809 |
| | A/G | 16 | 0.32 | 6 | 0.30 | 52 | 0.32 | 0.85 0.27-2.69 | 0.911 | 1.07 0.52-2.20 | 0.83 | 0.87 0.31-2.44 | 0.839 |
| | G/G | 5 | 0.10 | 1 | 0.05 | 13 | 0.08 | 0.39 0.04-3.75 | 0.398 | 1.44 0.46-4.54 | 0.772 | 0.57 0.07-4.75 | 1.000 |
| **TGF-β R1 rs334349* | G/G | 29 | 0.58 | 8 | 0.40 | 101 | 0.63 | 0.48 0.17-1.39 | 0.158 | 0.82 0.43-1.57 | 0.618 | 0.39 0.15-1.02 | 0.057 |
| | G/A | 18 | 0.36 | 7 | 0.35 | 48 | 0.3 | 1.39 0.41-4.71 | 0.882 | 1.37 0.68-2.78 | 0.61 | 1.84 0.63-5.42 | 0.615 |
| | A/A | 3 | 0.06 | 5 | 0.25 | 12 | 0.07 | 7.09 1.31-38.48 | 0.019 | 0.81 0.21-3.18 | 1.000 | 5.48 1.52-19.7 | 0.044 |
| ***TGF-β R2 rs4522809* | A/A | 33 | 0.66 | 15 | 0.75 | 55 | 0.34 | 1.54 0.48-4.98 | 0.444 | 3.74 1.91-7.31 | 0.0001 | 5.78 1.99-16.7 | 0.001 |
| | A/G | 12 | 0.24 | 2 | 0.10 | 78 | 0.48 | 0.35 0.07-1.81 | 0.145 | 0.24 0.11-0.52 | 0.0002 | 0.09 0.02-0.40 | 0.0004 |
| | G/G | 5 | 0.10 | 3 | 0.03 | 28 | 0.17 | 1.31 0.27-6.35 | 0.567 | 0.53 0.19-1.45 | 0.268 | 0.84 0.23-3.06 | 1.000 |
| ****VEGF-A rs3025039* | C/C | 33 | 0.66 | 10 | 0.50 | 116 | 0.72 | 0.52 0.86-8.27 | 0.279 | 0.75 0.38-1.48 | 0.478 | 0.39 0.15-1.00 | 0.068 |
| | C/T | 10 | 0.20 | 8 | 0.40 | 40 | 0.25 | 2.67 1.00-13.00 | 0.129 | 0.99 0.44-2.23 | 0.091 | 2.59 0.94-7.17 | 0.085 |
| | T/T | 7 | 0.14 | 2 | 0.10 | 5 | 0.03 | 1.20 0.20-7.13 | 0.745 | 5.08 1.53-16.8 | 0.0085 | 4.89 0.82-29.1 | 0.074 |

* *rs334349*: SCC Vs BCC recessive model 6.20 (1.25-30.81) p=0.022; SCC Vs Controls recessive model 4.31 (1.32-14.08) p=0.024

** *rs4522809*: BCC Vs Controls dominant model 0.24 (0.12-0.49) p<0.0001; SCC Vs Controls dominant model 0.15 (0.05-0.45) p=0.0002

*** *rs3025039*: BCC Vs Controls recessive model 3.94 (1.15-13.49) p<0.0001; SCC Vs Controls dominant model 2.86 (1.09-7.50) p=0.034

**Table S4**. Analysis of frequencies of 31 pseudo-haplotype (p-Hp) containing major or minor alleles of *TGF-β* gene family and *VEGF-A* SNPs, in non-melanocytic skin cancer (NMSC) patients and controls (CTRL)

| p-Hp | *TGF-β1 rs1800471 G/C* | *TGF-β2 rs900 A/T* | *TGF-βR1 rs334348 A/G* | *TGF-βR1 rs334349 G/A* | *TGF-βR2 rs4522809 A/G* | *VEGF-A rs3025039 C/T* | CTRL | NMSC | OR (95%CI) | *p* |
|---|---|---|---|---|---|---|---|---|---|---|
| 1 | G | A | A | G | A | C | 0.218 | 0.282 | 1.44 (0.76-2.73) | 0.313 |
| 2 | G | A | A | G | G | C | 0.150 | 0.028 | 0.17 (0.04-0.73) | 0.0061 |
| 3 | G | T | A | G | G | C | 0.106 | 0.047 | 0.33 (0.10-1.17) | 0.0893 |
| 4 | G | A | G | A | A | C | 0.076 | 0.062 | 0.75 (0.23-2.42) | 0.788 |
| 5 | G | T | A | G | A | C | 0.071 | 0.096 | 1.51 (0.56-4.09) | 0.429 |
| 6 | G | A | G | G | A | C | 0.042 | 0.000 | 0.15 (0.01-2.60) | 0.105 |
| 7 | G | T | A | G | A | T | 0.041 | 0.025 | 0.65 (0.13-3.20) | 0.727 |
| 8 | G | A | A | A | A | C | 0.028 | 0.016 | 0.45 (0.05-3.94) | 0.671 |
| 9 | G | A | G | A | G | C | 0.026 | 0.008 | 0.57 (0.06-5.18) | 1.000 |
| 10 | C | A | A | G | A | C | 0.023 | 0.013 | 0.56 (0.06-5.19) | 1.00 |
| 11 | G | T | G | A | G | C | 0.022 | 0.000 | 0.24 (0.01-4.67) | 0.317 |
| 12 | G | A | A | G | G | T | 0.028 | 0.000 | 0.25 (0.01-4.68) | 0.317 |
| 13 | G | A | G | G | A | T | 0.021 | 0.007 | 0.76 (0.08-7.47) | 1.000 |
| 14 | G | A | A | G | A | T | 0.010 | 0.124 | 11.7 (2.46-55.9) | 0.0005 |
| 15 | G | T | A | A | G | C | 0.010 | 0.015 | 1.15 (0.10-12.9) | 1.000 |
| 16 | G | T | G | A | G | T | 0.010 | 0.016 | 1.15 (0.11-12.9) | 1.000 |
| 17 | C | A | A | G | A | T | 0.010 | 0.007 | 0.45 (0.02-9.54) | 1.000 |
| 18 | C | A | A | G | G | C | 0.015 | 0.000 | 0.46 (0.02-9.56) | 1.000 |
| 19 | C | A | A | G | G | T | 0.012 | 0.000 | 0.45 (0.02-9.55) | 1.000 |
| 20 | G | T | G | G | G | C | 0,011 | 0.000 | 0.45 (0.02-9.55) | 1.000 |
| 21 | C | A | A | A | G | C | 0.005 | 0.021 | 2.33 (0.14-37.6) | 0.515 |
| 22 | G | A | A | A | G | T | 0.008 | 0.015 | 2.32 (0.14-37.6) | 0.515 |
| 23 | G | A | G | G | G | C | 0.000 | 0.020 | 6.97 (0.28-173) | 0.303 |
| 24 | G | T | G | A | A | C | 0.000 | 0.058 | 21.8 (1.16-411) | 0.0079 |
| 16 | G | T | A | G | G | T | 0.000 | 0.016 | 6.97 (0.28-173) | 0.303 |
| 25 | G | T | A | A | A | T | 0.000 | 0.015 | 6.97 (0.28-173) | 0.303 |
| 26 | G | A | G | A | A | T | 0.003 | 0.020 | 6.97 (0.28-173) | 0.303 |
| 27 | C | A | G | A | A | C | 0.000 | 0.012 | 6.97 (0.28-173) | 0.303 |
| 28 | C | A | A | A | G | T | 0.003 | 0.011 | 6.97 (0.28-173) | 0.303 |
| 29 | C | A | G | A | G | C | 0.003 | 0.010 | 6.97 (0.28-173) | 0.303 |
| 30 | C | T | A | A | A | C | 0.000 | 0.014 | 6.97 (0.28-173) | 0.303 |
| 31 | C | A | G | G | A | C | 0.000 | 0.014 | 6.97 (0.28-173) | 0.303 |
